# A novel cell‐free DNA methylation‐based model improves the early detection of colorectal cancer

**DOI:** 10.1002/1878-0261.12942

**Published:** 2021-03-25

**Authors:** Xianrui Wu, Yunfeng Zhang, Tuo Hu, Xiaowen He, Yifeng Zou, Qiling Deng, Jia Ke, Lei Lian, Xiaosheng He, Dezhi Zhao, Xuyu Cai, Zhiwei Chen, Xiaojian Wu, Jian‐Bing Fan, Feng Gao, Ping Lan

**Affiliations:** ^1^ Department of Colorectal Surgery The Sixth Affiliated Hospital Sun Yat‐sen University Guangzhou China; ^2^ Guangdong Provincial Key Laboratory of Colorectal and Pelvic Floor Diseases The Sixth Affiliated Hospital Sun Yat‐sen University Guangzhou China; ^3^ Bioland Laboratory (Guangzhou Regenerative Medicine and Health Guangdong Laboratory) Guangzhou China; ^4^ AnchorDx Medical Co. Ltd Guangzhou China; ^5^ AnchorDx, Inc. Fremont CA USA; ^6^ Department of Pathology School of Basic Medical Science Southern Medical University Guangzhou China

**Keywords:** advanced adenoma, cell‐free DNA, colorectal cancer, early detection, methylation, sequencing

## Abstract

Screening for early‐stage disease is vital for reducing colorectal cancer (CRC)‐related mortality. Methylation of circulating tumor DNA has been previously used for various types of cancer screening. A novel cell‐free DNA (cfDNA) methylation‐based model which can improve the early detection of CRC is warranted. For our study, we collected 313 tissue and 577 plasma samples from patients with CRC, advanced adenoma (AA), non‐AA and healthy controls. After quality control, 187 tissue DNA samples (91 non‐malignant tissue from CRC patients, 26 AA and 70 CRC) and 489 plasma cfDNA samples were selected for targeted DNA methylation sequencing. We further developed a cfDNA methylation model based on 11 methylation biomarkers for CRC detection in the training cohort (area under curve [AUC] = 0.90 (0.85–0.94]) and verified the model in the validation cohort (AUC = 0.92 [0.88–0.96]). The cfDNA methylation model robustly detected patients pre‐diagnosed with early‐stage CRC (AUC = 0.90 [0.86–0.95]) or AA (AUC = 0.85 [0.78–0.91]). Here we established and validated a non‐invasive cfDNA methylation model based on 11 DNA methylation biomarkers for the detection of early‐stage CRC and AA. The utilization of the model in clinical practice may contribute to the early diagnosis of CRC.

## Graphical abstract

Cell‐free DNA (cfDNA) methylation is promising for colorectal cancer (CRC) screening. In this study, after DNA methylation sequencing of tissue and plasma samples, we established a cfDNA methylation model based on 11 DNA methylation biomarkers for the detection of early‐stage CRC and advanced adenoma. The utilization of the model may contribute to the non‐invasive early diagnosis of CRC.

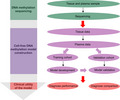

**Abbreviations**
AA: advanced adenoma
AUC: area under curve
AUROC: area under receiver operating characteristic curve
BHP: Benjamini–Hochberg procedure
CA19‐9: carbohydrate antigen 19‐9
CEA: carcino‐embryonic antigen
cfDNA: cell‐free DNA
CpG: cytosine‐phosphoric‐guanine
CRC: colorectal cancer
ctDNA: circulating tumor DNA
FFPE: formalin‐fixed paraffin‐embedded
FIT: fecal immunological test
gFOBT: guaiac‐based fecal occult blood test
LASSO: Least Absolute Shrinkage and Selection Operator
mt‐sDNA: multi‐target stool DNA
NAA: non‐advanced adenoma
NGS: next‐generation sequencing
PCM: percentage of co‐methylated reads
QC: quality control
SD: standard deviation

## 1 Introduction

Colorectal cancer (CRC) is the third most common malignant neoplasm globally [1]. Despite improvements in treatment, the prognosis of CRC patients with advanced TNM stage remains poor. The 5‐year survival rate of stage IV CRC patients is 14% [2], in contrast to 91% for stage I CRC patients [3]. Therefore, screening for early‐stage CRC is one of the key strategies for reducing CRC‐associated mortality. Currently, the approaches for CRC screening can mainly be separated into two types, invasive colonoscopy and non‐invasive stool‐based CRC screening, such as guaiac‐based fecal occult blood test (gFOBT), fecal immunological test (FIT) and multi‐target stool DNA (mt‐sDNA) test [4]. The compliance rate of colonoscopy is fairly low due to its invasiveness, high cost and the requirement for extensive bowel preparation [5]. The mt‐sDNA test has been criticized for its relatively high false‐positive rate, which sometimes leads to unnecessary treatment [6]. Although gFOBT and FIT are much more cost‐effective and convenient than colonoscopy, they have a relatively low sensitivity for detecting advanced adenoma (AA) and early‐stage CRC [7]. Therefore, a non‐invasive CRC screening test with high sensitivity and specificity is urgently needed.

DNA methylation is one of the epigenetic mechanisms that cells use to regulate gene expression [8]. It is well recognized that aberrant hypermethylation of cytosine‐phosphoric‐guanine (CpG) islands in tumor suppressor genes can result in transcriptional silencing and carcinogenesis [9]. Hypermethylation of tumor suppressor genes has been found to be an early event in many cancers [10, 11, 12, 13]. Moreover, aberrant methylation is dynamic and potentially reversible, making it a potential target for treatment [14]. Therefore, DNA methylation pattern holds a profound potential as a biomarker in cancer screening and monitoring.

Circulating tumor DNA (ctDNA) consists of extracellular nucleic acid fragments that are released into the blood via necrosis, apoptosis or active DNA secretion by tumor cells [15]. The quantity of cell‐free DNA (cfDNA) has been reported to be higher in several tumors, especially in those patients with advanced cancer stage, than in healthy individuals [16, 17, 18]. The amount of cfDNA was shown to be related to tumor size and clinical stage [19, 20]. Moreover, it has been reported that methylation levels of cfDNA in plasma are consistent with those in the primary tumor [21, 22, 23]. These findings suggest that alterations to the cfDNA methylation signature might be able to serve as ideal biomarkers for non‐invasive cancer screening and diagnosis [24].

In this study, we aimed to evaluate the potential value of cfDNA methylation pattern as a biomarker for the screening and diagnosis of CRC. We developed a novel CRC‐specific cfDNA methylation model using high‐throughput targeted DNA methylation sequencing. The CRC‐specific cfDNA methylation model was generated and further refined using a cohort of plasma cfDNA samples. An independent validation cohort was used to validate the robustness and accuracy of this model.

## 2 Materials and methods

### 2.1 Patient enrollment and sample collection

The DNA samples used in this study were obtained from fresh‐frozen tissues, formalin‐fixed paraffin‐embedded (FFPE) tissues, and plasma. All of the specimens were collected at The Sixth Affiliated Hospital of Sun Yat‐sen University from August 2016 to May 2018. This study was approved by the ethics committee of The Sixth Affiliated Hospital of Sun Yat‐sen University (2016ZSLYEC‐056). The experiments were undertaken with the understanding and written consent of each subject and the study methodologies conformed to the standards set by the Declaration of Helsinki.

#### 2.1.1 Tissue samples

The tumor tissues and the corresponding adjacent normal tissues were derived from patients receiving CRC resection. The adjacent normal tissues were collected from the normal intestine, which were more than 5 cm away from the primary tumor. The tissue samples of AA were obtained from FFPE specimens from The Sixth Affiliated Hospital of Sun Yat‐sen University.

#### 2.1.2 Plasma samples

A 10‐mL aliquot of blood were drawn from healthy controls or treatment‐naive patients using BD Vacutainer® EDTA Tubes (Becton, Dickinson and Company, Plymouth, UK, Cat# 367525) and the plasma was immediately separated within 2 h after blood draw and was stored at −80 °C for a median of 9 days (range: 1–35 days) until DNA isolation and subsequent assays. The resultant plasma volume ranged from 2.0 to 3.2 mL. The healthy controls consisted of patients with benign anorectal diseases, such as hemorrhoids, anal fissures and perianal fistulae. To be included in this study, the healthy controls had to meet the following inclusion criteria: (1) age older than 18 years old, (2) no other significant medical history, such as cancer or chronic diseases, (c) willingness before the blood draw to have a colonoscopy which was normal.

### 2.2 Isolation of tissue genomic DNA and plasma cell‐free DNA

Tissue genomic DNA was isolated from fresh‐frozen and FFPE tissue samples using the Qiagen DNeasy Blood & Tissue Kit (Qiagen, Hilden, Germany, Cat#: 69504) and the QIAamp DNA FFPE Tissue Kit (Qiagen, Cat# 56404), respectively.

Cell‐free DNA was isolated from plasma using the Bioo NextPrep‐Mag™ cfDNA Isolation Kit (Bioo Scientific, Austin, TX, USA, Cat# NOVA‐3825). Repeated freezing and thawing of plasma were avoided to prevent cfDNA degradation. The concentration and quality of cfDNA were determined using the Qubit™ dsDNA HS Assay Kit (Thermo Fisher Scientific, Eugene, OR, USA, Cat# Q32854) and the Agilent High Sensitivity DNA Kit (Agilent, Waldbrann, Germany, Cat# 5067‐4626) on a 2100 Bioanalyzer Instrument (Agilent), which assessed the size distribution of cfDNA. The cfDNA with yield > 3 ng and without overt genomic DNA contamination was used for sequencing library construction.

### 2.3 Bisulfite conversion

Bisulfite conversion was performed using the Zymo Lightning Conversion Reagent (Zymo Research, Irvine, CA, USA, Cat# D5031) according to the manufacturer’s protocols. For tissue samples, 2 μg of genomic DNA was fragmented into ~ 200‐bp fragments (peak size) by an M220 Focused‐ultrasonicator (Covaris, Inc., Boston, MA, USA) following the manufacturer’s instructions, and 800 ng of purified fragmented genomic DNA was then used for the following bisulfite conversion. After bisulfite conversion, the purified bisulfite‐converted DNA was quantified at A260 by NanoDrop (Thermo Fisher Scientific). Then, 100 and 150 ng of the bisulfite‐converted products were applied for library preparation for fresh‐frozen and FFPE tissue samples, respectively. For plasma samples, the recommended input of cfDNA for bisulfite conversion was 10 ng. If cfDNA yield was between 3 and 10 ng, all the purified cfDNA was used for bisulfite conversion. After bisulfite conversion, we used all the bisulfite‐converted cfDNA for library preparation without DNA quantification to avoid cfDNA loss. Following DNA bisulfite conversion, the bisulfite‐converted DNA was run through a Zymo‐Spin™ IC Column (Zymo Research, Cat# D5031), washed and desulfonated, and then eluted twice using the M‐Elution buffer to a final volume of 17 μL.

### 2.4 AnchorIRIS™ pre‐library construction

AnchorIRIS™ (Guangzhou, Guangdong) pre‐hyb library construction was performed using AnchorDx EpiVisio^TM^ Methylation Library Prep Kit (AnchorDx, Guangzhou, China, Cat# A0UX00019) and AnchorDx EpiVisio^TM^ Indexing PCR Kit (AnchorDx, Cat# A2DX00025). Following the procedure of end pair reparation, 3′ end adaptor ligation, and amplification of reverse complement DNA (Liang *et al*. [25]), the amplified DNA was purified using 1 : 6 Agencourt AMPure XP Magnetic Beads (Beckman Coulter, Brea, CA, USA, Cat# A63882). After 3′ end adaptor ligation of reverse complement DNA and indexing PCR (i5 and i7; Liang *et al*. [25]), the amplified pre‐libraries were subsequently purified using XP Magnetic Beads. Pre‐hyb libraries containing more than 800 ng DNA were used for target enrichment assay.

### 2.5 AnchorIRIS™ target enrichment

Target Enrichment was performed using AnchorDx EpiVisio^TM^ Target Enrichment Kit (AnchorDx, Cat# A0UX00031) and methylation panels, AnchorDx PanMet V1 or V2. A total of 1000 ng of DNA containing up to four pre‐hyb libraries were pooled for target enrichment using AnchorDx PanMet V1 or V2 methylation panel. AnchorDx PanMet V2 included 12 624 pre‐selected regions which were enriched for cancer‐specific methylation and contained all the 9921 regions of AnchorDx PanMet V1. The total size of the genomic regions targeted by the AnchorDx PanMet V1 and V2 panel was 563 272 and 733 057 bp, which covered 45 566 and 55 369 CpG sites, respectively. Probe hybridization, purification and final PCR amplification were carried out according to the protocols from Liang [25].

### 2.6 DNA methylation level calculation

Enriched libraries were sequenced by Illumina HiSeq X Ten Sequencing System. Percentage of co‐methylated reads (PCM) was calculated by the analysis pipeline developed by Liang [25].

$$\mathrm{PCM}=\frac{\#\mathrm{co}‐\mathrm{methylated}\;\mathrm{reads}\;\mathrm{of}\;a\;\mathrm{region}}{\#\;\mathrm{all}\;\mathrm{mapped}\;\mathrm{reads}\;\mathrm{with}\;\mathrm{at}\;\mathrm{least}\;3\;\mathrm{CpGs}\;\mathrm{in}\;\mathrm{the}\;\mathrm{region}}.$$

Reads having at least three methylated CpGs within a sliding window of five CpGs were designated as co‐methylated reads and used for subsequent analysis of methylation pattern and predictive modeling of malignant/normal states of patient samples. Log_2_ PCM was used for the model construction to optimize the model’s performance and stability.

### 2.7 Statistical analysis

#### 2.7.1 CRC‐specific cfDNA methylation biomarkers for diagnostic analysis

With the AnchorDx PanMet methylation panels, we first performed a differential methylation analysis in normal, AA and CRC tissue samples using Wilcoxon signed‐rank test, where the *P*‐value for each methylation biomarker was corrected by multiple testing through the Benjamini–Hochberg procedure (BHP) to control the false‐discovery rate at a significance level of 0.05. We also calculated the distinguishing power by the area under receiver operating characteristic curve (AUROC) and absolute methylation change for each biomarker. CRC‐specific DNA methylation biomarkers were identified using the following criteria:

- significant difference between CRC and normal tissue samples (adjusted *P* < 0.05) with relatively large absolute change (> 0.2);
- significant difference between AA and normal tissue samples (adjusted *P* < 0.05) with relatively large absolute change (> 0.2);
- same trend for CRC and AA compared with normal controls.

We also added DNA methylation biomarkers that were significantly different between AA and CRC to improve the potential differentiation power. Consequently, a total of 667 CRC‐specific DNA methylation biomarkers were obtained in this tissue cohort analysis.

#### 2.7.2 Development and validation of the CRC cfDNA methylation screening model

After obtaining these 667 CRC‐specific DNA methylation biomarkers based on the tissue samples, we then analyzed the plasma samples, where we identified 545 biomarkers with differential DNA methylation levels in the plasma between CRC patients and healthy controls (*P* < 0.05). Then, the plasma cfDNA methylation dataset consisting of CRC patients and healthy controls was randomly split into a training and a validation cohort with matched gender, age and TNM stage. In the training cohort, 545 differentially expressed DNA methylation biomarkers were used for subsequent biomarker shrinkage and model construction. Least Absolute Shrinkage and Selection Operator (LASSO) was applied for variable selection. Lambda with minimum error estimated by 10‐fold cross‐validation was used. Eleven DNA methylation biomarkers were obtained for model building based on a binary prediction. Eventually, we constructed a logistic regression model using these 11 biomarkers as the covariate in the training cohort. A CRC diagnosis risk score was calculated by multiplying the unbiased coefficient estimate and the biomarker methylation value matrix in both the training and validation cohort. The predictability of the model was evaluated by AUROC, which calculated the proportion of concordant pairs among all pairs of observations. The model’s diagnosis performance for non‐AA (NAA), AA, and CRC patients was then evaluated. All data were shown as mean ± standard deviation (SD).

## 3 Results

### 3.1 Patient and sample characteristics

**Fig. 1:**
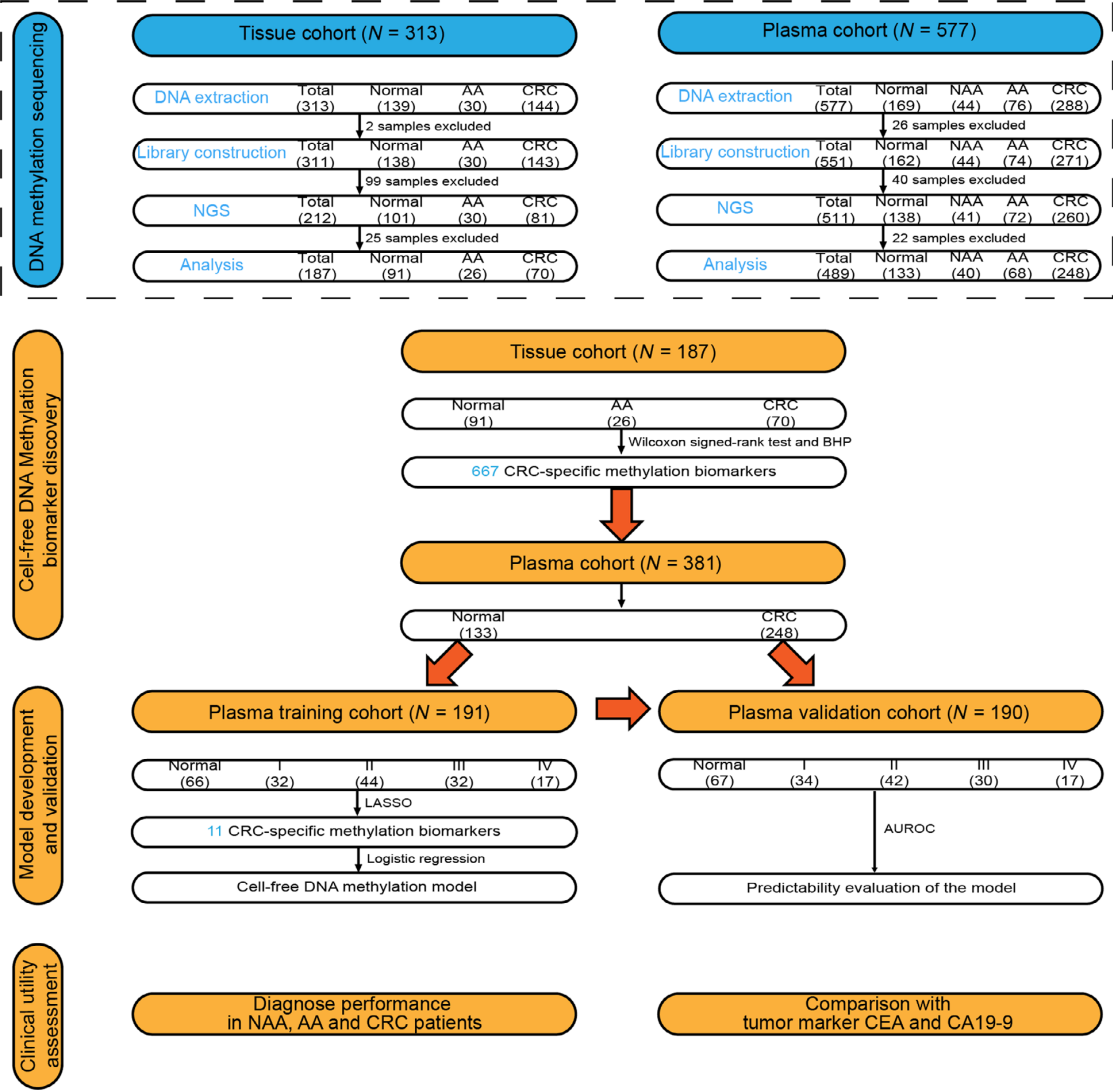
The study workflow chart. In the DNA methylation sequencing phase, 313 tissue samples (139 Normal, 30 AA and 144 CRC) were collected for NGS. Additionally, 577 plasma samples (169 Healthy controls, 44 NAA, 76 AA and 288 CRC) were collected for NGS. After DNA extraction, library construction and DNA methylation sequencing, 187 tissue samples and 489 plasma samples were eventually analyzed. Wilcoxon signed‐rank test and BHP were applied to screen the CRC‐specific methylation biomarkers in the tissue cohort, which led to the discovery of 667 DNA methylation biomarkers. In all, 133 normal plasma samples and 248 CRC plasma samples were randomly assigned to the training and validation cohort, respectively, and were then analyzed to further identify CRC‐specific methylation biomarkers from these 667 biomarkers. After LASSO selection, 11 CRC‐specific methylation biomarkers were obtained using the training cohort, which were then further confirmed using the validation cohort. Ultimately, the clinical value of the model was assessed by performing diagnostic tests in NAA, AA and CRC patients. The robustness of the model in the management of CRC was evaluated by comparison with CEA and CA19‐9. I, CRC stage I; II, CRC stage II; III, CRC stage III; IV, CRC stage IV.

To characterize DNA methylation biomarkers which were specific to CRC, a total of 313 tissue samples were collected (139 normal, 30 AA, 144 CRC). After DNA extraction and AnchorIRIS™ library construction, 212 DNA samples passed quality control (QC) and were subsequently used for DNA methylation next‐generation sequencing (NGS), and 101 samples were excluded due to failure of DNA extraction QC (DNA yield and quality; *n* = 2) or low library yield (*n* = 99). A further 25 samples did not pass the sequencing QC metrics. Ultimately, 187 samples (91 normal, 26 AA, and 70 CRC) were analyzed for the discovery of DNA methylation biomarkers specific to CRC (Fig. 1).

To explore the clinical application of cfDNA methylation biomarkers for the detection of early‐stage CRC, 577 plasma samples (169 Normal , 44 NAA, 76 AA and 288 CRC) were collected for DNA methylation sequencing. All of the patients and healthy controls were treatment‐naive before the blood draw. After excluding 66 samples that did not pass DNA extraction QC (*n* = 26) or that had limited library yield (*n* = 40), a total of 511 samples were used for NGS. Finally, 489 plasma samples (133 Normal, 40 NAA, 68 AA and 248 CRC) that passed sequencing QC were subsequently analyzed for model development and validation (Fig. 1). Detailed information about sample exclusion is shown in Table S1. Clinical characteristics of the 489 patients or healthy controls with plasma analyzed are listed in Table 1.

**Table 1:** The demographic and clinical characteristics of the healthy controls and patients in the plasma cohort. IA, inapplicable.

Characteristics	Normal	NAA	AA	CRC
Total (*n*)	133	40	68	248
Gender				
Male, *n* (%)	76 (57.14)	25 (62.50)	43 (63.24)	143 (57.66)
Female, *n* (%)	57 (42.86)	15 (37.50)	25 (36.76)	105 (42.34)
Age (years)				
Mean	44	56	59	60
Range	18–78	38–86	23–86	24–89
Stage				
I, *n* (%)	IA	IA	IA	66 (26.61)
II, *n* (%)	IA	IA	IA	86 (34.68)
III, *n* (%)	IA	IA	IA	62 (25.00)
IV, *n* (%)	IA	IA	IA	34 (13.71)

### 3.2 Measurement of cell‐free DNA concentration

**Fig. 2:**
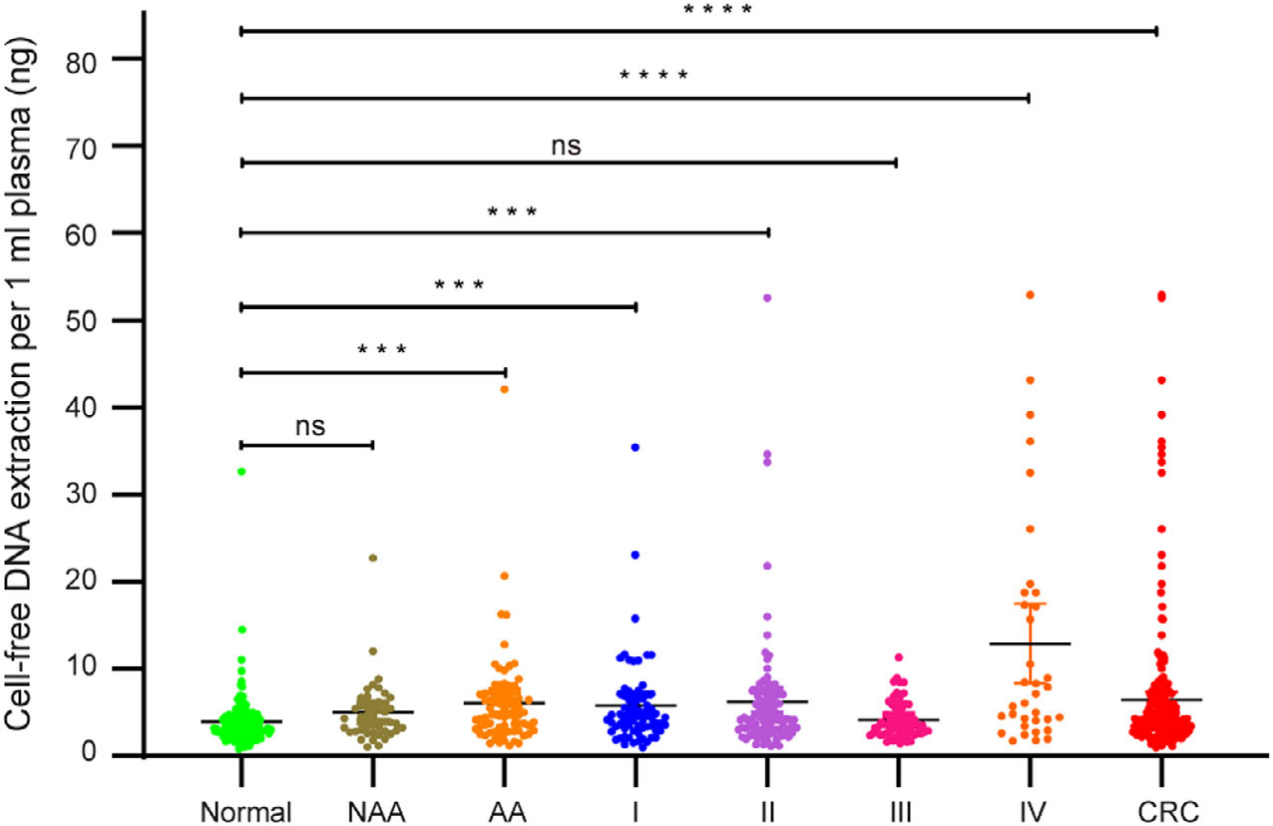
The cfDNA extraction analysis in healthy controls, NAA, AA and CRC patients. A total of 551 (162 Healthy controls [Normal], 44 NAA, 74 AA, 69 CRC stage I [I], 97 CRC stage II [II], 70 CRC stage III [III], 35 CRC stage IV [IV]) cfDNA extraction QC‐qualified samples were measured and compared for the cfDNA concentration (paired Student’s *t*‐test). Data are shown as mean ± SD; ns, not significant; ***P < 0.001; ****P < 0.0001.

As shown in Fig. 1, the concentration of 551 cfDNA samples (162 Normal, 44 NAA, 74 AA and 271 CRC) that passed DNA extraction QC was measured using a Qubit® fluorescent dye method. The concentration of cfDNA from CRC and AA samples was significantly higher than that from healthy control samples (Fig. 2). CRC and AA patients yielded a mean cfDNA concentration of 6.43 ng ± 0.45 and 6.08 ng ± 0.65 per 1 mL plasma, respectively, whereas healthy controls had a mean concentration of 3.94 ng ± 0.24 per 1 mL plasma (Table S2). Overall, the cfDNA concentration in CRC and AA patients was higher than that of healthy controls.

### 3.3 Characterization of methylation biomarkers specific to CRC

**Fig. 3:**
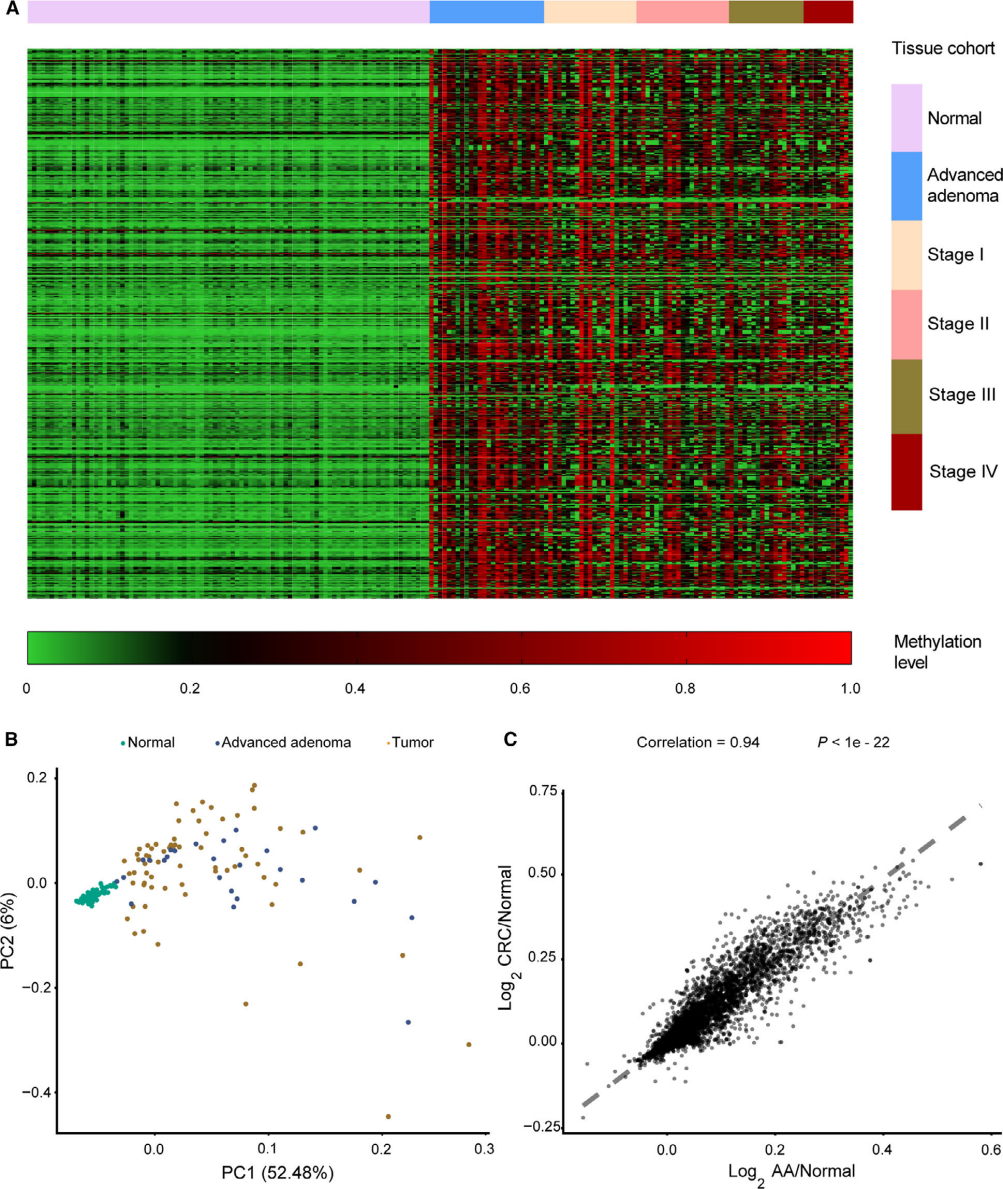
Characterization of the tissue DNA methylation landscape. (A) Unsupervised hierarchical clustering of the 667 CRC‐specific DNA methylation biomarkers in 187 tissue samples. (B) Principal component analysis of CRC, AA and Normal cohort. (C) Correlation of the methylation pattern between CRC and AA group. The mean methylation level was calculated based on 9921 sequenced biomarkers. The values plotted were generated by dividing PCM of the Normal cohort followed by log_2_ transformation.

After analyzing the tissue cohort’s sequencing data using Wilcoxon signed‐rank test and BHP‐adjust method, 667 CRC‐specific DNA methylation biomarkers that distinguished CRC and AA from normal tissue samples were identified (Fig. 3A). The correlation analysis result of these 667 biomarkers is shown in Fig. S1. These biomarkers were found to be distributed frequently in the intronic (28.34%) and promoter (25.19%) region of the genome (Table S3). The methylation levels in the normal cohort differed significantly from those in the AA and CRC cohort (Fig. 3B). Moreover, the levels of DNA methylation biomarkers in the AA group, which were calculated by dividing the normal group’s PCM and Log_2_ transformed values, were similar to those in the malignant group, which suggests that DNA methylation might be an early epigenetic event in CRC (Fig. 3C).

### 3.4 Development and validation of the cfDNA methylation model for CRC detection

**Fig. 4:**
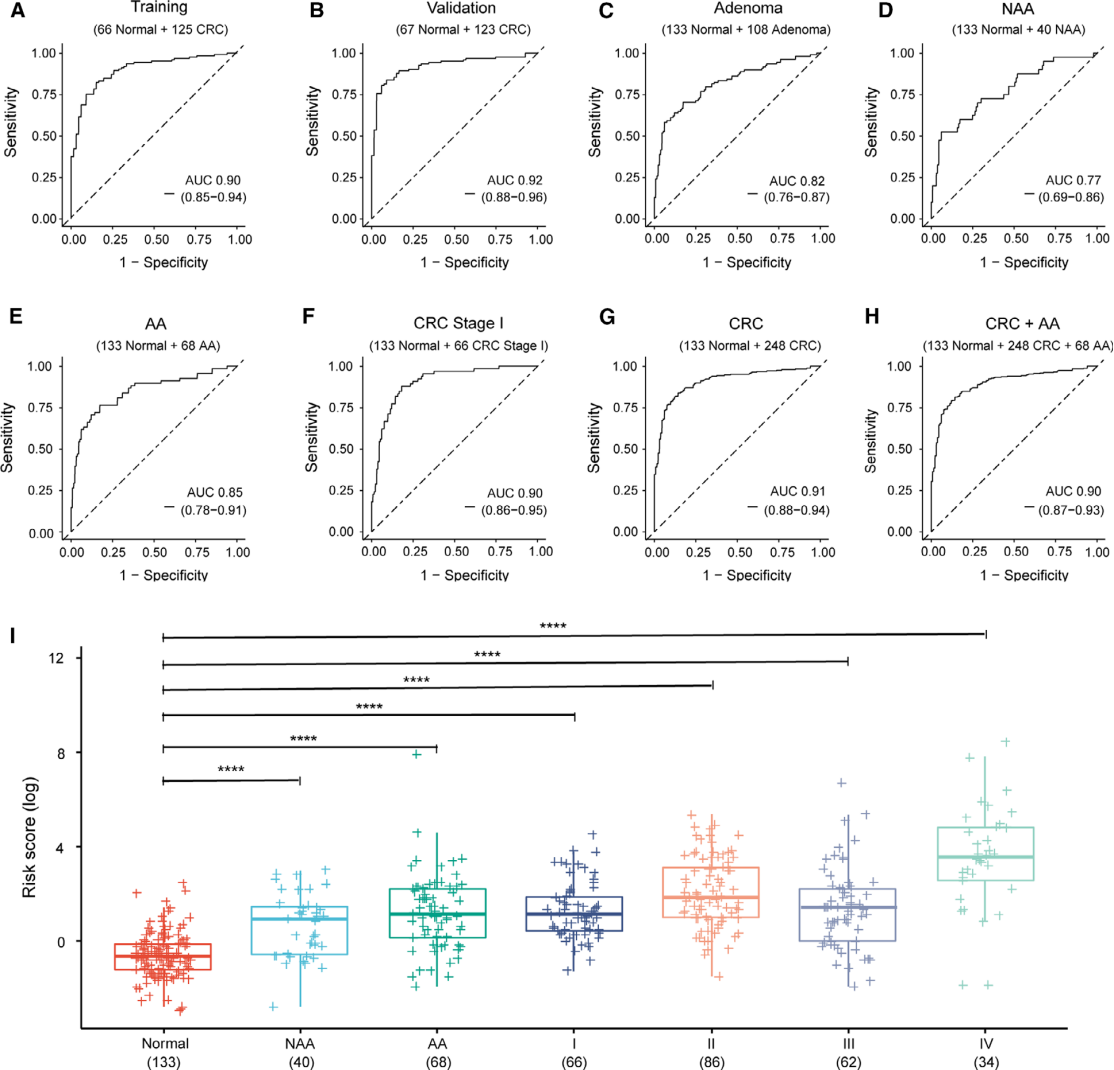
The performance and risk score of the cfDNA methylation model in detecting adenoma and CRC patients. (A,B) AUC of the model was 0.90 (0.85–0.94) and 0.92 (0.88–0.96) in the training and validation cohort, respectively. (C–E) When applied to the diagnosis of adenoma patients, the model achieved an AUC of 0.82 (0.76–0.87), 0.77 (0.69–0.86) and 0.85 (0.78–0.91) in adenoma, NAA and AA patients, respectively. (F) AUC of the model in the detection of CRC stage I was 0.90 (0.86–0.95; *n* = 199). (G) The model performed robustly in diagnosing CRC patients, which achieved an AUC of 0.91 (0.88–0.94; *n* = 381). (H) The overall AUC of the model was 0.90 (0.87–0.93) in the detection of CRC and AA cohort (*n* = 449). (I) The risk score of the model in healthy controls (Normal) and in patients with NAA, AA and CRC stage I–IV (*n* = 489, paired Student’s *t*‐test). The error bars indicate confidence interval; ****P < 0.0001.

After the discovery of 667 CRC‐specific DNA methylation biomarkers in the tissue cohort, we further analyzed the methylation of these 667 biomarkers in the plasma cohort (Fig. 1). In all, 133 normal and 248 CRC plasma samples were randomly assigned to the training and validation cohort, respectively, after matching for gender, age and TNM stage. The clinical characteristics of the training and validation cohort are shown in Table S4. The LASSO method was applied to shrink the number of CRC‐specific DNA methylation biomarkers in the training cohort. Eleven age‐independent methylation biomarkers were eventually obtained (Table S5, Fig. S2). The cfDNA methylation model with these 11 biomarkers was then constructed using logistic regression. With a risk score threshold of 0.58 defined by Youden’s indexing in the training cohort, the cfDNA methylation model yielded a sensitivity of 82.4% and a specificity of 84.8% for CRC detection in the training cohort, and a sensitivity of 84.6% and a specificity of 86.6% in the validation cohort, respectively (Table S6). This model could distinguish CRC patients from healthy controls in both the training (AUC = 0.90) and the validation cohort (AUC = 0.92; Fig. 4A,B). We then evaluated the performance of this model in separating NAA, AA and stage I CRC patients from healthy controls, and found that the AUC was 0.77, 0.85 and 0.90, respectively (Fig. 4D–F). Furthermore, the risk score that was calculated based on the 11 DNA methylation biomarkers was demonstrated to be significantly higher in NAA, AA and CRC patients than in healthy controls (Fig. 4I). These data indicated that the cfDNA methylation model could serve as a robust and non‐invasive method for CRC detection.

### 3.5 Comparison of clinical utility in CRC detection between cfDNA methylation model and conventional tumor biomarker

**Fig. 5:**
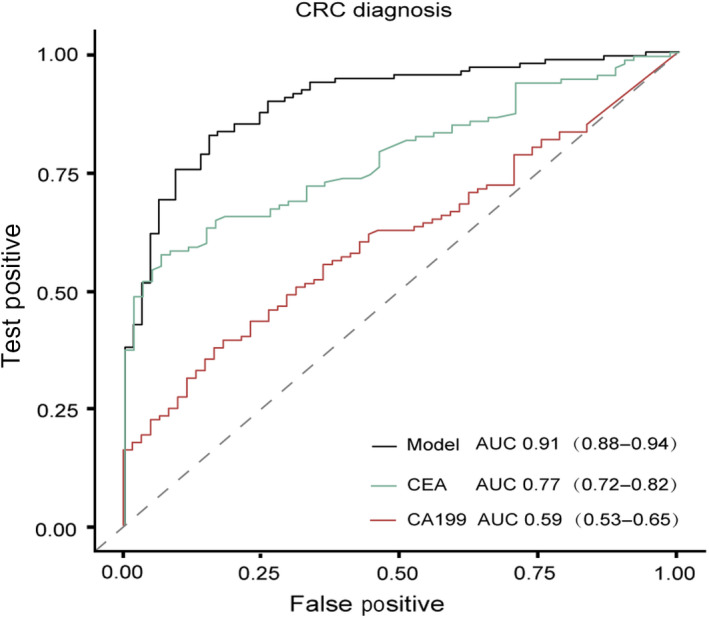
Comparison of CRC diagnostic performance between the cfDNA methylation model and the tumor biomarker CEA and CA19‐9. AUC in detecting CRC of the cfDNA methylation model, CEA and CA19‐9 was 0.91 (0.88–0.94), 0.77 (0.72–0.82) and 0.59 (0.53–0.65), respectively.

Currently, carcino‐embryonic antigen (CEA) and carbohydrate antigen 19‐9 (CA19‐9) are among the most commonly used blood tumor biomarkers for therapy monitoring in CRC. In addition, the abnormal increase of CEA and CA19‐9 leads to the suspicion of CRC in clinical practice. A comparison in the performance in CRC detection was carried out between the cfDNA methylation model and CEA and CA19‐9 monitoring. The cfDNA methylation model was shown to be superior to CEA and CA19‐9 in CRC detection in terms of both sensitivity and specificity (AUC 0.91 versus 0.77 and 0.59, respectively, Fig. 5, Table 2). In addition, cfDNA methylation model as opposed to CEA and CA19‐9 monitoring, correlated well with AA and tumor stage (Table 2), showcasing the advantage of the cfDNA methylation model over CEA and CA19‐9 in the early detection of CRC.

**Table 2:** AUC, sensitivity, specificity and accuracy of the cfDNA methylation model, CEA and CA19‐9 in disease diagnosis.

Characteristics	AA	CRC Stage I	CRC Stage II	CRC
AUC				
Model	0.85	0.90	0.94	0.91
CEA	0.64	0.68	0.78	0.77
CA19‐9	0.54	0.48	0.60	0.59
Sensitivity				
Model	76.5%	87.9%	83.7%	83.9%
CEA	40.0%	55.6%	58.8%	63.6%
CA19‐9	57.9%	31.6%	39.5%	29.8%
Specificity				
Model	82.7%	82.0%	91.7%	85.7%
CEA	82.6%	81.0%	89.3%	81.0%
CA19‐9	55.4%	81.8%	81.0%	90.1%
Accuracy				
Model	80.6%	83.9%	88.6%	84.5%
CEA	67.7%	66.9%	76.7%	69.3%
CA19‐9	56.2%	53.8%	63.8%	49.6%

## 4 Discussion

Detection at the early stage is pivotal for the successful treatment of various cancer types, including CRC [26]. Colonoscopy accompanied by tissue biopsy remains the gold standard in the diagnosis of CRC [27]. However, colonoscopy is less than perfect for the purpose of CRC screening due to its invasiveness, high cost, time consumption and unpleasant examination experience [28]. Furthermore, the accuracy of detecting early‐stage CRC patients by endoscopy can vary significantly between different endoscopists [29]. Therefore, an accurate, robust and non‐invasive test is highly desirable for CRC screening.

DNA methylation aberration usually occurs early in the progression of many tumors, suggesting that detection of altered DNA methylation patterns could be a promising strategy in cancer screening [30]. Moreover, the genomic profile of ctDNA was shown to share features with that of concurrent tumor in the same cancer patient, which has important implications for non‐invasive cancer screening [31]. Progress in high‐throughput sequencing technology and the availability of multi‐omics have contributed to developing diagnostic tools for the early detection of cancers [32, 33]. However, to our knowledge, the methylation profile of different stages of CRC progression, which comprises normal tissue, AA and various CRC stages, is rarely available, even though the risk of progression from AA to CRC has been commonly acknowledged [34]. Intending to construct a cfDNA methylation model for the early detection of CRC, we first analyzed DNA methylation status of tissue samples from CRC, AA and normal mucosa, which helped identify DNA methylation biomarkers that could distinguish CRC tumor from normal tissue. Moreover, we evaluated the capability of a blood‐based cfDNA methylation test for early detection of CRC. A diagnostic model based on 11 cfDNA methylation biomarkers showed high performance in distinguishing CRC from normal individuals. The overall sensitivity of CRC detection was 83.9% in the validation cohort at a specificity of 85.7%. The model, especially, achieved high sensitivities on AA (76.5%) and stage I CRC (87.9%), which is critical for detecting CRC in the stages with curative treatments.

At present, several blood‐based DNA methylation biomarkers have been assessed for early detection of CRC. The Epi proColon assay (SEPT9) yielded an overall sensitivity of 48.2% at a specificity of 91.5% for CRC detection in a prospective clinical trial of 7941 asymptomatic individuals [35]. In a cohort of 2105 individuals, a two‐biomarker blood test (BCAT1/IKZF1) identified 66% of CRC at a specificity of 95% [36]. A single methylation biomarker, cg10673833, was demonstrated to be superior to other previous reported methylation biomarkers in CRC detection, with a sensitivity and specificity of 89.7% and 86.8%, respectively [37].

The benefit of all screening tests for reducing CRC morbidity and mortality relies on the test performance in detecting stage I CRC and AA [38]. The sensitivity of SEPT9 (Epi proColon assay) was 35.0% and 11.2% for detecting stage I CRC and AA, respectively [35], although a recent study reported improved performance by analyzing multiple regions of this gene [39]. The BCAT1/IKZF1 methylation test identified 38% of stage I CRC but only 6% of AA [36]. The single methylation biomarker, cg10673833, had a sensitivity of 33.3% against CRC advanced precancerous leision which included AA [37]. A pan‐cancer detection test based on cfDNA methylation profiling yielded a sensitivity of ~ 25% for stage I CRC [40]. Although a head‐to‐head comparison of the performance between our 11‐biomarker CRC diagnostic model and other screening tests has not been made, this approach showed superior sensitivities of 87.9% in stage I CRC and 76.5% in AA. Nevertheless, further verification using colonoscopy remains recommended in cases of positive results as suggested by the 11 methylation biomarkers, due to the false‐positive results. Overall, these results suggested that this model could serve as a promising screening test for non‐invasive detection of CRC at an early and curable stage, which needs further validation in real clinical settings.

There are certain limitations associated with this study. First, the plasma samples were retrospectively collected and less than optimal, as most of the CRC patients enrolled in the plasma cohort were symptomatic and were older than healthy controls, which might influence the model’s clinical accuracy.

Secondly, the specimens used in this study were collected from a single institution despite its large sample size. Therefore, the robustness of the cfDNA methylation model should be further validated in a prospective, multi‐center trial.

Thirdly, the cfDNA methylation model was constructed based on hypermethylated regions in CRC tissue. However, some CRC‐specific methylation regions may not be hypermethylated [41] and might have been excluded from our biomarker discovery step. Therefore, it is plausible that a plasma‐based discovery step is needed to identify other cfDNA signatures that have not been included in the current panel.

Fourthly, the clinical utility of the cfDNA methylation model in CRC detection was compared with CEA and CA19‐9, which are not used as CRC screening biomarkers in clinical practice due to their low sensitivity. Hence, it is necessary to conduct a prospective study to compare our model with more appropriate non‐invasive CRC screening tests, such as FIT.

Lastly, samples of sessile serrated poly (SSP), a type of premalignant lesion of CRC [42], should be included in future studies to test the suitability of our CRC risk model for detecting such lesion.

## 5 Conclusions

We established a promising and non‐invasive cfDNA methylation model for the detection of CRC, especially early‐stage CRC and AA. The diagnostic value of this model was validated in an independent cohort, highlighting its promising application for early detection of CRC.

## Conflict of interest

J‐BF, DZZ, XYC and ZC are/were employees of AnchorDx Medical Co., Ltd. or AnchorDx, Inc. All other authors declare no competing financial interest.

## Author contributions

PL, FG and J‐BF designed and supervised the study and revised the manuscript. XRW, YFZ, XYC and ZC developed the methodology including analyzing and organizing data. TH, XWH, YFZ, QLD, JK, LL, XSH, DZZ and XJW were responsible for acquiring and managing patients. YFZ, XRW, FG, ZC, JBF and PL wrote and reviewed the manuscript. All authors read and approved the final manuscript.

### Peer Review

The peer review history for this article is available at https://publons.com/publon/10.1002/1878‐0261.12942.

## Supporting information

**Fig. S1.** The correlation analysis of the 667 CRC‐specific DNA methylation biomarkers.
**Fig. S2.** The correlation analysis between age and 11 CRC‐specific DNA methylation biomarkers.
**Table S1.** The reasons for tissue and plasma sample exclusion.
**Table S2.** The cfDNA extraction quantity in CRC, AA, NAA patients and healthy controls.
**Table S3.** The distribution of the 667 CRC‐specific DNA methylation biomarkers in the genome.
**Table S4.** The demographic and clinical characteristics of the training and validation cohort.
**Table S5.** The genomic characteristics of the 11 DNA methylation biomarkers.
**Table S6.** The AUC, sensitivity, specificity and accuracy of the cfDNA methylation model in diagnosis of CRC.

## Data Availability

The datasets which were used and analyzed during the current study are available from the corresponding author on reasonable request.
