# Supplementary material for: A novel cell‐free DNA methylation‐based model improves the early detection of colorectal cancer
Source: Mol Oncol. 2021 Mar 25;15(10):2702–14. doi: 10.1002/1878-0261.12942 (PMC8486566; doi:10.1002/1878-0261.12942)

**Supplementary Materials**

**1.** **Supplementary Table.**

**1.1** **Supplementary Table S1. The reasons for tissue and plasma sample exclusion.**

| Characteristic | QC Detail | QC-failed (Tissue) | Left (Tissue) | QC-failed (Plasma) | Left (Plasma) |
| --- | --- | --- | --- | --- | --- |
| Enrollment | Starting | 0 | 313 | 0 | 577 |
| DNA extraction | DNA yield and quality | 2 | 311 | 26 | 551 |
| Library construction | Library yield | 99 | 212 | 40 | 511 |
| Sequence | CHH%/CHG%, coverage, etc. | 25 | 187 | 22 | 489 |

Note: CHH%/CHG%, “C” means Cytosine, “H” means Adenine/ Cytosine/Guanine CHG, “G” means Guanine, CHH%/CHG% represents all of the cytosine in non CpG sites. CHH%/CHG% in mammal is usually under 1%, thus, it can be used for the examination of bisulfite conversion efficiency.

**1.2** **Supplementary Table S2. The cfDNA extraction quantity in CRC, AA, NAA patients and healthy controls.**

| Characteristic | Normal | NAA | AA | I | II | III | IV | CRC |
| --- | --- | --- | --- | --- | --- | --- | --- | --- |
| Case number | 162 | 44 | 74 | 69 | 97 | 70 | 35 | 271 |
| Minimum (ng/ml) | 0.79 | 1.01 | 1.17 | 0.99 | 1.11 | 1.44 | 1.76 | 0.99 |
| Maximum (ng/ml) | 32.59 | 22.67 | 42.02 | 35.39 | 52.50 | 11.29 | 52.89 | 52.89 |
| Mean (ng/ml) | 3.94 | 5.01 | 6.08 | 5.80 | 6.20 | 4.12 | 12.88 | 6.43 |
| Standard Deviation | 0.24 | 0.54 | 0.65 | 0.62 | 0.72 | 0.24 | 2.25 | 0.45 |
| P value  (compared to Normal) | IA | 0.05 | 0.0002 | 0.0009 | 0.0005 | 0.6600 | <0.0001 | <0.0001 |

Note: cfDNA, cell-free DNA; CRC, Colorectal cancer; AA, Advanced adenoma; NAA, Non-advanced adenomas; I, Colorectal cancer stage I; II, Colorectal cancer stage II; III, Colorectal cancer stage III; IV, Colorectal cancer stage IV; IA, inapplicable.

**1.3 Supplementary Table S3. The distribution of the 667 CRC-specific DNA methylation biomarkers in the genome.**

| Location | Number of biomarkers | Percentage (%) |
| --- | --- | --- |
| Downstream | 1 | 0.15 |
| Exonic | 42 | 6.30 |
| Intergenic | 86 | 12.89 |
| Intronic | 189 | 28.34 |
| ncRNA_exonic | 26 | 3.90 |
| ncRNA_intronic | 53 | 7.95 |
| Upstream | 168 | 25.19 |
| UTR3 | 6 | 0.90 |
| UTR5 | 96 | 14.39 |
| Total | 667 | 100 |

**1.4 Supplementary Table S4. The demographic and clinical characteristics of the training and validation cohort.**

| Characteristics | Training cohort | Validation cohort |
| --- | --- | --- |
| Total (n) | 191 | 190 |
| Gender |  |  |
| Male-no. (%) | 110(57.59) | 109(57.37) |
| Female-no. (%) | 81(42.41) | 81(42.63) |
| Age (years) |  |  |
| Mean | 55 | 54 |
| Range | 22-89 | 18-89 |
| Stage |  |  |
| Normal | 66(34.56) | 67(35.26) |
| I-no. (%) | 32(16.75) | 34(17.89) |
| II-no. (%) | 44(23.04) | 42(22.11) |
| III-no. (%) | 32(16.75) | 30(15.79) |
| IV-no. (%) | 17(8.90) | 17(8.95) |

Note: I, Colorectal cancer stage I; II, Colorectal cancer stage II; III, Colorectal cancer stage III; IV, Colorectal cancer stage IV.

**1.5 Supplementary Table S5. The genomic characteristics of the 11 DNA methylation biomarkers.**

| Name | Chromosome | Relation to CpG Island | Regulatory Feature Group |
| --- | --- | --- | --- |
| cg00310855 | chr11:65,111,621-65,111,742 | Island | Promoter Associated |
| cg01857475 | chr12:104303355-104303476 | N Shore | Promoter Associated |
| cg01922936 | chr5:100903253-100903374 | Island | Promoter Associated |
| cg11320449 | chr5:181052562-181052683 | Island | Unclassified |
| cg11407741 | chr10:8037328-8037449 | S Shore | Unclassified Cell type specific |
| cg11596863 | chr12:112575351-112575472 | Island | IA |
| cg15020425 | chr11:69818888-69819009 | Island | Unclassified Cell type specific |
| cg22329423 | chr20:21509874-21509995 | Island | Unclassified |
| cg24733262 | chr15:79091242-79091363 | Island | IA |
| cg25300584 | chr20:24469108-24469229 | N Shore | IA |
| cg26337020 | chr5:100903258-100903379 | Island | Promoter Associated |

Note: IA, inapplicable.

**1.6 Supplementary Table S6. The AUC, sensitivity, specificity, and accuracy of the cfDNA methylation model in diagnosis of CRC.**

| Characteristics | Training cohort (95% CI) | Validation cohort (95% CI) |
| --- | --- | --- |
| Threshold | 0.58 (0.41-0.74) | 0.58 (0.41-0.74) |
| AUC | 0.90 (0.86-0.94) | 0.92 (0.88-0.96) |
| Sensitivity | 82.4% (69.6%-94.4%) | 84.6% (77.1%-89.9%) |
| Specificity | 84.8% (72.7%-97.0%) | 86.6% (76.4%-92.8%) |
| Accuracy | 83.2% (77.5%-89.5%) | 85.9% (81.2%-91.7%) |

**2.** **Supplementary Figure.**

**2.1 Supplementary Figure S1. The correlation analysis of the 667 CRC-specific DNA methylation biomarkers.**


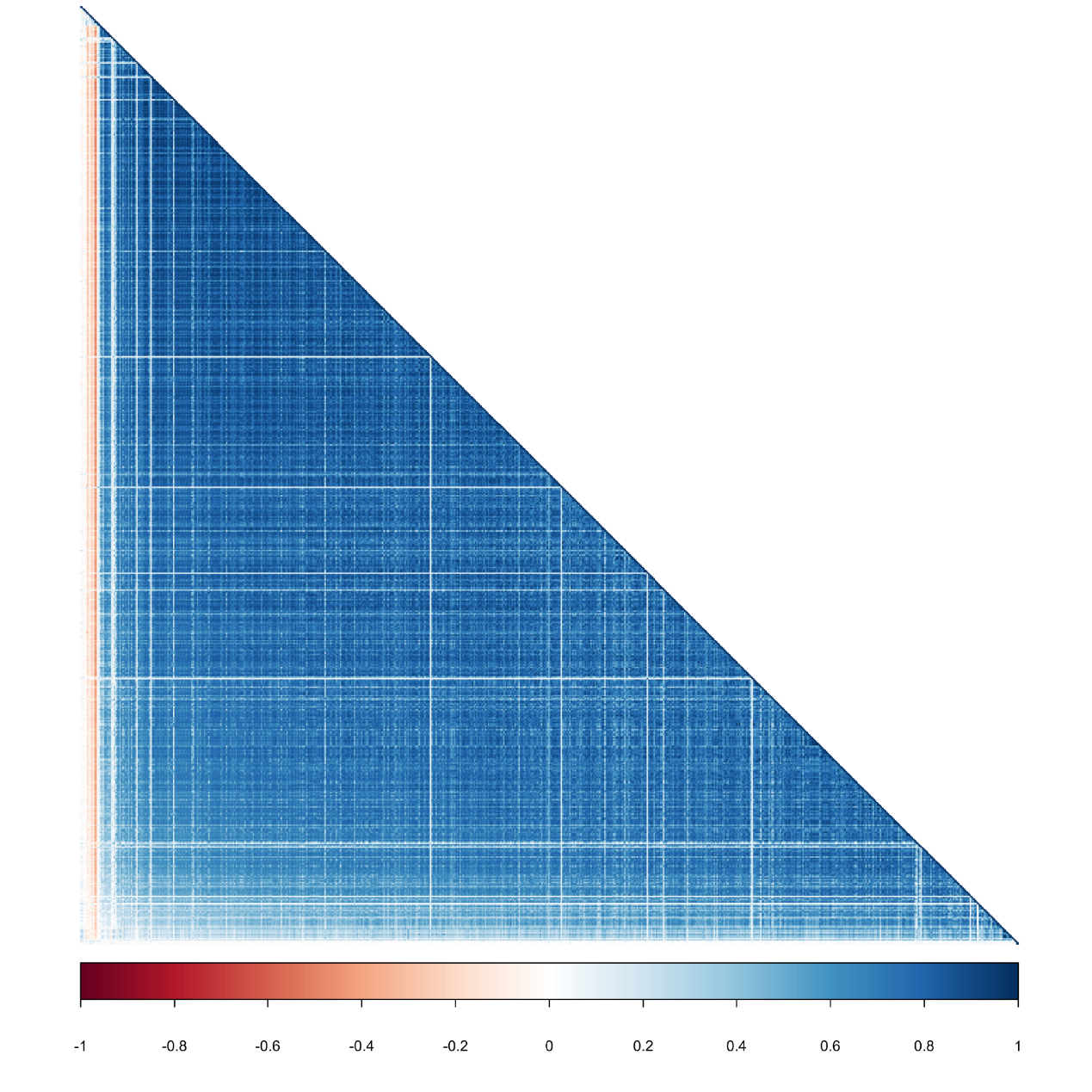


**2.2 Supplementary Figure S2. The correlation analysis between age and 11 CRC-specific DNA methylation biomarkers.** (A-K). CRC-specific DNA methylation biomarker of cg00310855, cg01857475, cg01922936, cg11320449, cg11407741, cg11596863, cg15020425, cg22329423, cg24733262, cg25300584, and cg26337020 was age-independent (P > 0.05 or Correlation < 0.2). **p < 0.01.


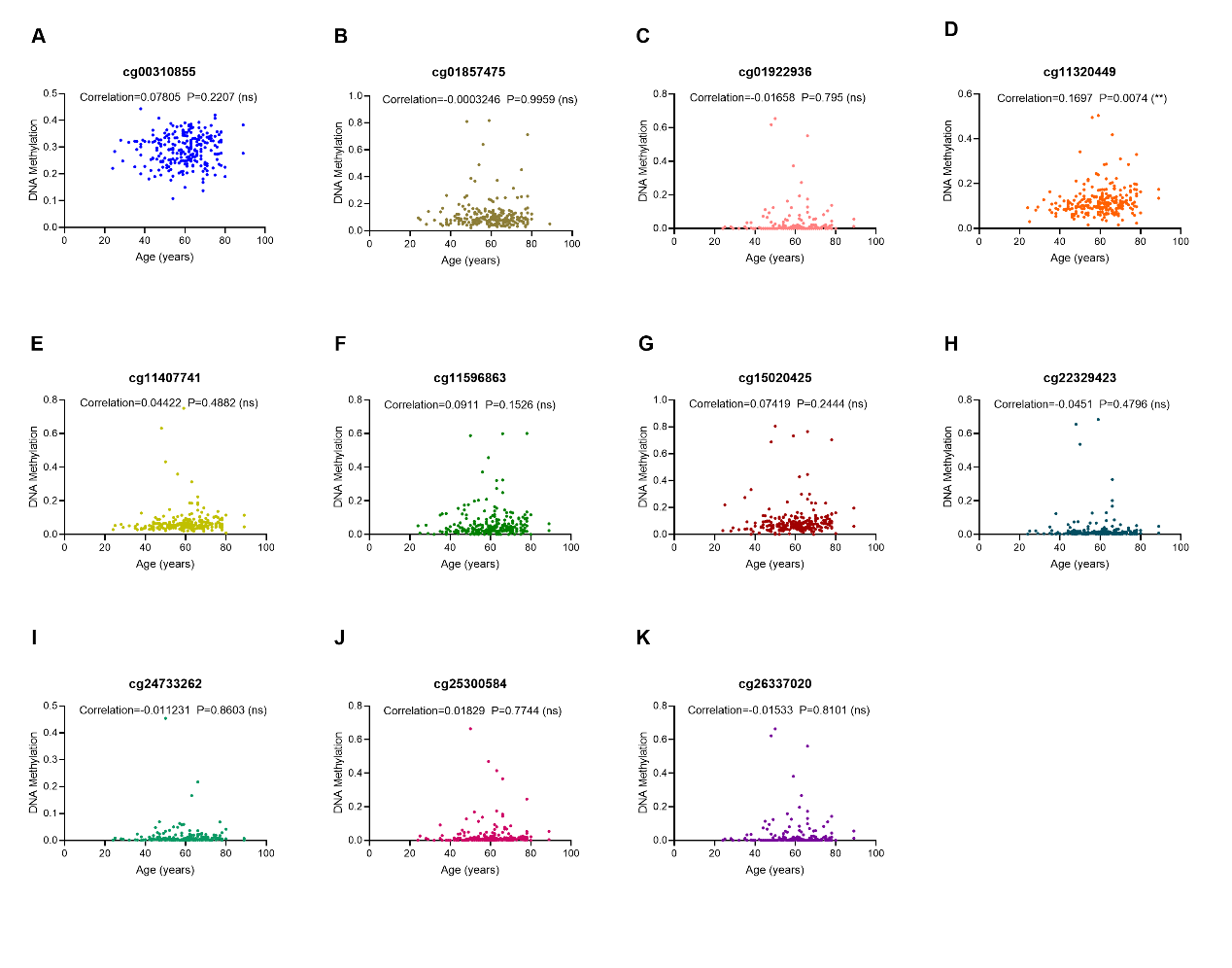

Supplement: Supplementary file 1 — Fig. S1. The correlation analysis of the 667 CRC‐specific DNA methylation biomarkers. Fig. S2. The correlation analysis between age and 11 CRC‐specific DNA methylation biomarkers. Table S1. The reasons for tissue and plasma sample exclusion. Table S2. The cfDNA extraction quantity in CRC, AA, NAA patients and healthy controls. Table S3. The distribution of the 667 CRC‐specific DNA methylation biomarkers in the genome. Table S4. The demographic and clinical characteristics of the training and validation cohort. Table S5. The genomic characteristics of the 11 DNA methylation biomarkers. Table S6. The AUC, sensitivity, specificity and accuracy of the cfDNA methylation model in diagnosis of CRC. [file MOL2-15-2702-s001.docx]
